# Self-Assembled Monolayers for Dental Implants

**DOI:** 10.1155/2018/4395460

**Published:** 2018-02-06

**Authors:** Sidónio C. Freitas, Alejandra Correa-Uribe, M. Cristina L. Martins, Alejandro Pelaez-Vargas

**Affiliations:** ^1^Faculty of Dentistry, Universidad Cooperativa de Colombia, Medellin, Colombia; ^2^Private Practice in Periodontology, Medellin, Colombia; ^3^Instituto de Investigação e Inovação em Saúde (I3S), Instituto de Engenharia Biomédica (INEB) and Instituto de Ciências Biomédicas Abel Salazar (ICBAS), Universidade do Porto, Porto, Portugal

## Abstract

Implant-based therapy is a mature approach to recover the health conditions of patients affected by edentulism. Thousands of dental implants are placed each year since their introduction in the 80s. However, implantology faces challenges that require more research strategies such as new support therapies for a world population with a continuous increase of life expectancy, to control periodontal status and new bioactive surfaces for implants. The present review is focused on self-assembled monolayers (SAMs) for dental implant materials as a nanoscale-processing approach to modify titanium surfaces. SAMs represent an easy, accurate, and precise approach to modify surface properties. These are stable, well-defined, and well-organized organic structures that allow to control the chemical properties of the interface at the molecular scale. The ability to control the composition and properties of SAMs precisely through synthesis (i.e., the synthetic chemistry of organic compounds with a wide range of functional groups is well established and in general very simple, being commercially available), combined with the simple methods to pattern their functional groups on complex geometry appliances, makes them a good system for fundamental studies regarding the interaction between surfaces, proteins, and cells, as well as to engineering surfaces in order to develop new biomaterials.

## 1. Introduction

The World Health Organization points out two entities of bacterial origin, caries and periodontitis, which are the most disseminated diseases in human, and both are associated with frequent surgical procedures [1]. These infectious diseases and other noninfectious diseases such as dentoalveolar trauma and congenital absences are the main causes of edentulism. Several preventives and educational programs are used to avoid or reduce the role of these infectious diseases in the early loss of teeth. However, dentoalveolar trauma has been increased due to human activities such as extreme and contact sports [2–4].

Partial edentulism produces deleterious effects on the balance of the cranio-cervico-facial system because it may affect soft and hard tissues. Intra-arch changes include missing of interproximal contacts, misalignment, diastema, rotation, inclination, periodontal defects, impactation, and mesial drift displacement. Inter-arch changes have been described as occlusal collapse, premature occlusal contact, infraocclusion, and altered vertical dimension. These changes are synergic, increasing the bruxism, muscle parafunction, teeth wear, ATM symptom, otologic pain, and craniocervical position [5].

**Figure 1:**
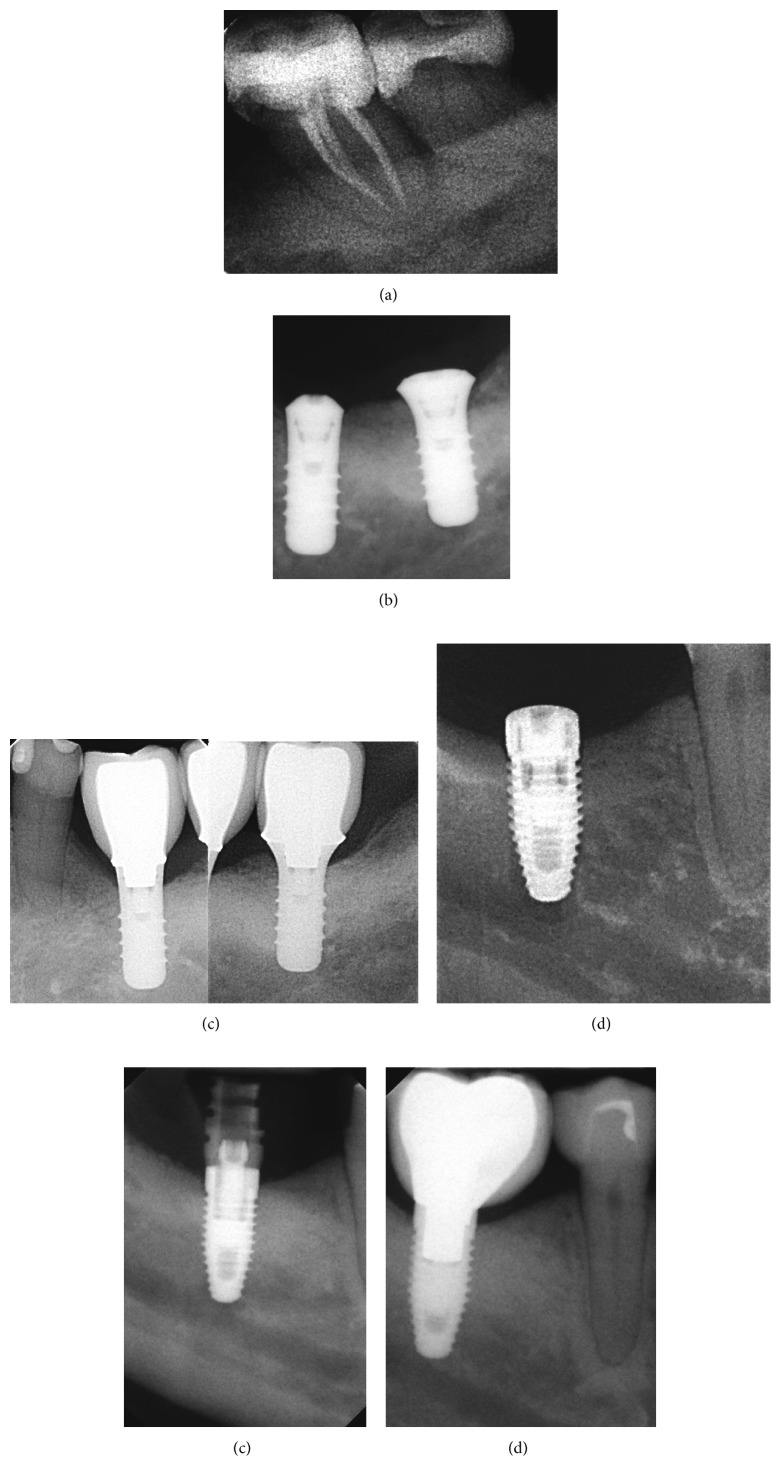
Successful cases. Patient 1: initially, an important reduction of support tissues was observed (a), and periapical X-ray obtained during the surgical procedure (b) and 5 years of follow-up (c). Patient 2: periapical X-ray obtained during implant surgery (d), restorative processing (e), and 5 years of follow-up (f).

Recovering aesthetics and function is possible using orthodontics when space closing is an alternative feasible while in other cases are required surgical and oral rehabilitation procedures such as implant-supported or removable prostheses. Titanium implant-based therapy appears as the “gold standard,” with a record of ∼95% survival rates reported after 5 years [6]. However, other concepts have been introduced such as “the success” (Figure 1), which is different from long-term survival because it is focused on integral evaluation in terms of aesthetic, function, and biological response, with less than 0.2 mm of apical migration [7].

The implantology history shows two different phases defined as pre-osseointegration and post-osseointegration eras. During the pre-osseointegration era, blade- and plate-form implants were developed using cobalt-chromium-molybdenum and different stainless steel types. However, a limited long-term success was achieved. Post-osseointegration era started with Branemark's research a decade before that his research were presented to scientific community, followed by Albrektsson et al. studies to verify clinically the osseointegration of implants [8]. The osseointegration concept was defined as a biological phenomenon involving direct contact between bone and Ti surfaces, opening a new paradigm of therapy. A wide revision about this topic is available [8, 9].

Implant therapy has high levels of predictability in a short term presenting few contraindications to restore partial and full edentulism. Many factors have been evaluated to predict the short-term effect including surgical stability, individual inflammatory response, periodontal covering, and blood clot formation. However, long-term predictability has been associated with several aspects such as implant-related designs, surgical procedures, anatomic and osseous conditions, systemic diseases, habits like bruxism, prosthetic design, susceptibility, periodontal status, oral microenvironment, native or augmented bone, two-stage or immediate loading, and adherence to support therapy. A poor prognosis is observed in patients with an insufficient quality and/or quantity of bone; patients exhibiting poor quality of bone (type IV) in the posterior area of the maxilla had a 35% implant failure while patients with types I, II, and III showed only 3% failure [10].

The long-term success of an implant (Figure 1) largely depends on the balance between occlusal equilibrium, osseointegration, and epithelial/connective tissue attachment. A complete sealing of the soft tissue protects the newly formed bone from bacterial metabolic products originated in the biofilm formed around implant [11].

Several animal and *in vitro* studies have shown similar epithelial and connective structures between the gingiva and the peri-implant mucosa. The outer surface of the peri-implant mucosa is aligned by a continuous stratified keratinized oral epithelium with a junctional epithelium attached to the Ti surface by a basal lamina and hemidesmosomes. The nonkeratinized junctional epithelium has only a few cell layers in the apical portion and is separated from the alveolar bone by a collagen-rich connective tissue. This 3–4 mm biological barrier, which is formed regardless of the original mucosal thickness, protects the osseointegration zone from factors released by the plaque and the oral cavity [12]. The main differences between the soft tissues around natural teeth and those around implants are the collagen fibre orientation, which run parallel from the implant surface to the crest bone, the low number of fibroblasts, the reduced vascularization revealed as scar tissue, and the loss of the irrigation system of the periodontal ligament [13].

**Figure 2:**
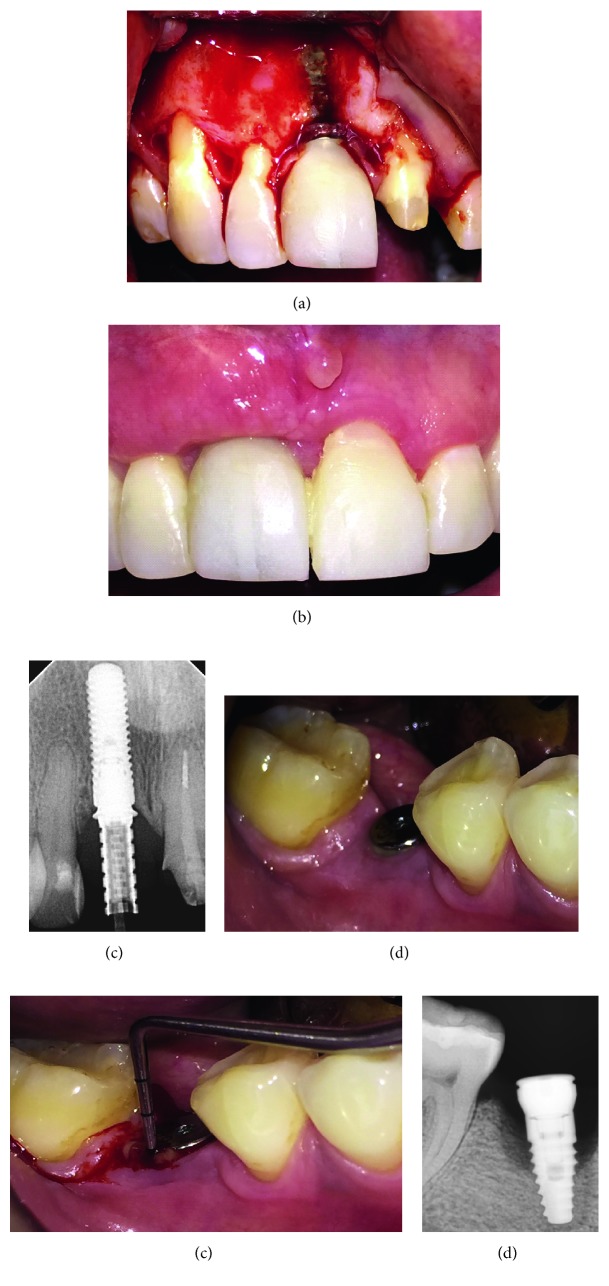
Failure cases. Patient 3: exploratory surgical procedure for peri-implantitis (a), soft tissues after a bone graft healing (b), and periapical X-ray after 4 months (c). Patient 4: intraoral photography of implant (d), periodontal probing (e), and periapical X-ray showing vertical bone loss around implant (f).

An osseointegrated implant is not exempt from failure and complications. They are classified as biological, mechanical, material surface, iatrogenic, and patient-related failures. Mobility is a sign of implant failure and can be presented as rotational, lateral or horizontal, and axial or vertical [14]. There are different terms in the literature associated with biological implant failures like peri-implant diseases, mucositis, and peri-implantitis (Figure 2), where the first two are reversible inflammatory reactions around a functioning implant while peri-implantitis is a chronic inflammation with a loss of the supporting tissues around the implant induced by bacterial colonization and facilitated by the implant/abutment gap and by the chemistry and surface roughness of screw and restorative components [15].

Bacteria colonize and develop biofilms on the transmucosal abutment of osseointegrated dental implants. Like the gingival crevice around the natural tooth, the peri-implant mucosa covering the alveolar bone is closely adapted to the implant. In partially edentulous subjects, the developing microbiota around implants closely resembles the microflora of the natural teeth [12]. In addition to the dark-pigmented, Gram-negative anaerobic rods, other bacteria are associated with peri-implant infections (*Tannerella forsythia*, *Fusobacterium nucleatum*, *Campylobacter rectus*, *Parvimonas micra*, and *Prevotella intermedia*) [16], and eventually with *Staphylococcus* spp. and *Candida* spp. [17].

The surface texture of dental implants affects the rate of osseointegration [18] and biomechanical fixation. Surface roughness may be classified as “macro,” “micro,” and “nano” sized topologies. The “macro” ranges from millimetres to 10 *μ*m and is directly related to implant geometry with threaded screws and macroporous coatings helping the primary stability of the implants during the early phases of implantation. However, high surface roughness may increase peri-implantitis risk compared with moderate roughness (1-2 *μ*m) within “micro” range (1–10 *μ*m), maximizing bone/implant interlocking. Surface profiles in the “nano” range play an important role in protein adsorption and osteoblast adhesion and, thus, in osseointegration [19], but no reproducible surface roughness is currently clinically available.

This review firstly presents a brief overview of different coating strategies to increase the osseointegration of titanium and is followed by a detailed description of self-assembled monolayers as a nanoscale approach to modify dental implant surfaces.

## 2. Biofunctionalization Strategies Available for Dental Implants

All surface modification strategies described below aimed to improve the long-term clinical survival and success of those dental biomaterials without altering their bulk properties (e.g., mechanical and nontoxicity). These coating strategies are mainly focused to increase the osseointegration than to reduce the bacterial colonization.

Bioactive surfaces have been developed to improve the osseointegration of bone with dental materials like titanium through coating strategies with immobilized biomolecules such as cell adhesive peptides having the Arg-Gly-Asp (RGD) sequence or bone morphogenetic proteins (BMPs) that play important roles in bone formation *in vivo* [20], to promote the adhesion of bone cells (i.e., osteoblasts) and subsequent proliferation and mineralization activities [21, 22], to induce alkaline phosphatase activity in fibroblast [23] or the attachment of osteoblast [24].

Antiadhesive surfaces have been used to avoid/resist protein adsorption and microbial adhesion by immobilization or coating of synthetic polymers like poly(ethylene glycol) (PEG)/poly(ethylene oxide) (PEO) [25] and poly(methacrylic acid) [26] or natural polymers as chitosan [27].

Finally, antibacterial coatings have been applied using biocidal substances (e.g., antibiotics and antimicrobial peptides) through two systems: (a) a continuous release system, creating a local effect around the implant and (b) a permanent immobilization scheme, acting on local microorganisms that contact the surface.

Furthermore, one promising strategy to enhance tissue integration is to develop a selective biointeractive surface that simultaneously enhances bone cell function while decreasing bacterial adhesion [20, 22, 28].

A resume of surface modification methods and effects is presented in Table 1.

## 3. Basic Aspects of Self-Assembled Monolayers (SAMs)

SAMs are spontaneously formed by solution deposition through the immersion of an appropriate substrate into a solution of an active surfactant in an appropriate solvent (e.g., organic or aqueous) or by aerosol spraying or vapor deposition of the active organic compound onto the solid surface, being immersion the most popular and widely studied method for monolayer formation since it is the easiest and most inexpensive way to be applied to appliances with complex geometries [62–64]. In a typical procedure, freshly prepared or clean substrate is immersed in a dilute 1–10 mm solution of surfactant compound(s) in high purity solvent for 12–48 h at room temperature. After this period, the slides are withdrawn, rinsed with solvent, and dried under a stream of nitrogen [62].

**Figure 3:**
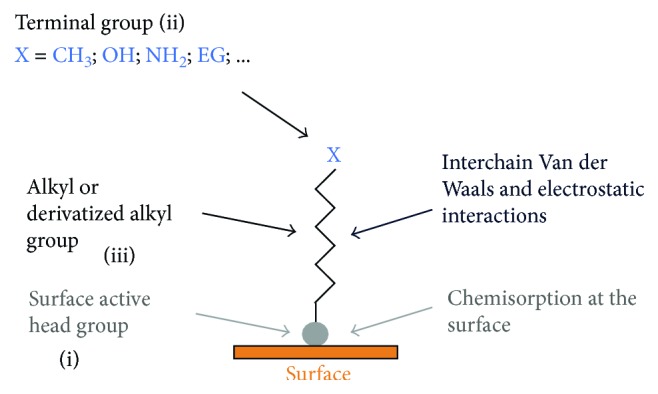
Schematic representation of a surfactant that can form a SAM.

The driving force for self-assembly is usually the specific interaction between the head group of the surfactant and the surface of the substrate. Most surfactants used for monolayer studies consist of three distinctive parts: (i) the surface active head group which binds strongly to the surface, (ii) the terminal group that is located at the monolayer surface and normally determines the interfacial properties of the assembly, and (iii) the alkane chain serves as a linker between the head and the terminal groups and facilitates the packing of the molecules in the monolayer with the Van der Waals interactions between adjacent methylene groups that orient and stabilize the monolayer (Figure 3) [62–66].

Considering that SAMs surface modifications are at nanoscale, physical and chemical characterizations appear as an important challenge to develop new market products based on this technology. Currently, several specialized surface analytical techniques are available to characterize SAMs for scientific approaches. The most commonly used techniques in routine SAMs characterization are as follows: ellipsometry, infrared reflection absorption spectroscopy (IRRAS), X-ray photoelectron spectroscopy (XPS), contact angle measurements, near edge X-ray absorption fine structure (NEXAFS), static time-of-flight secondary ion mass spectrometry (ToF-SIMS), surface imaging techniques such as scanning tunneling (STM) and atomic force microscopy (AFM), and electrochemical methods such as capacitance measurements (with cyclic voltammetry or impedance spectroscopy) and heterogeneous electron transfer (cyclic voltammetry). The general analytical capabilities of some of these techniques are presented in Table 2.

SAMs can be of different nature according to the surface described in Table 3.

The most widely studied and characterized class of SAMs is alkanethiols on gold, which have been used in model systems for various purposes, including corrosion resistance, protein adsorption, cell adhesion, and biosensors. Therefore, taking into account the focus of this chapter, a brief description of alkanethiols-SAMs on gold with bioactive osseointegration, antiadhesive, and antibacterial properties is presented below. (For more details on the structure and assembly, preparation, and characterization of gold-alkanethiol monolayers, see references [62, 66, 72]).

## 4. SAMs on Gold as Model Surface for Biomaterials

SAMs of alkanethiols on gold have been used as model surfaces for modulate cells adhesion, including osteoblasts and fibroblasts, by the immobilization of specific ligands or proteins such as RGD peptides and fibronectin [80–82].

Concerning antiadhesive surfaces, SAMs presenting oligomers of ethylene glycol, commonly prepared using the alkanethiols HS(CH_2_)_11_(OCH_2_CH_2_)_*n*_OH (EG_*n*_, *n* = 3–7 or OEG), resist the adsorption of several proteins and the adhesion of cells [83–91]. OEG-SAMs on gold present low adsorption of several blood proteins and blood cell adhesion as well as adhesion of the gastrointestinal bacterial species *H. pylori* as reported by us [86, 92–94]. This antiadhesive effect has been explained through theoretical and experimental research [95–99], indicating that water penetrates into the (EG)_*n*_OH layers of the SAMs forming a stable interfacial water layer, which prevents the direct contact between the underlying surface and the proteins and/cells. In addition, SAMs that comprise an OEG-terminated thiol with an alkanethiol terminated with either a biological ligand or a reactive site for linking to a biological ligand can present the ligands of interest in a structurally well-defined manner against a background that resists the nonspecific adsorption of other biomolecules or adhesion of cells. Moreover, the OEG terminal group also does not compromise receptor function either to promote the attachment and proliferation of eukaryotic cells as osteoblast and fibroblast for improving osseointegration or to avoid the adhesion and colonization of prokaryotic cells following an antibacterial strategy as described below [62].

**Figure 4:**
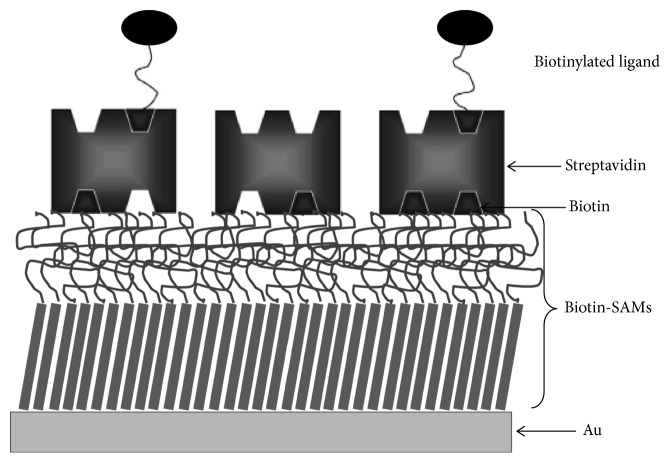
Schematic representation of mixed SAMs prepared with biotinylated alkanethiol (BAT) and triethylene glycol alkanelthiol (EG3) followed by streptavidin adsorption and ligand immobilization (not scale). Adapted from Freitas et al. [107].

Antibacterial SAMs strategies have been performed through the immobilization of biomolecules such as antibodies, antibiotics, and antimicrobial peptides by covalent or affinity binding. One of the strongest noncovalent receptor-ligand binding interactions known in nature is the biotin-avidin/streptavidin system, where both streptavidin and avidin have a very high degree of specificity and affinity to biotin (*K*_d_ = 10^−13^ M) with four equivalent sites for biotin [100]. This high binding affinity and selectivity, the symmetry of the biotin-binding pockets that are positioned in pairs at opposite faces of the protein, and the ease of functionalization of diverse biomolecules (e.g.,, antibodies, peptides, and nucleotides) with biotin make the streptavidin-biotin system extremely useful in a wide range of biotechnological applications such as in affinity separations, in diagnostic assays, and for “tagging” of molecules for imaging or delivery of therapeutics [101]. This affinity system is also applied to immobilize biomolecules/ligands onto SAMs surfaces by using alkanethiol terminally functionalized with a biotin moiety (Figure 4) [102–104]. These SAMs can bind streptavidin or avidin with high coverage, specificity, and activity in such a way to expose two of its binding sites away from the surface. Secondary molecules modified with biotin can then be rapidly and conveniently immobilized on these streptavidin/avidin-activated surfaces with minimal impact on their biological activity (e.g., specificity) [105]. Biotin-containing SAMs (BTMs) have been used in both antiadhesive and antibacterial surface strategies as discussed in Table 4. However, although this system is successful and widely used, it shares the disadvantages of most immobilization schemes requiring chemical modification of a protein: (i) chemical modification may lead to denaturation or loss of activity and (ii) the presence of multiple sites on the protein available for modification results in loss of control over its orientation after immobilization [106].

On the contrary, immobilization by covalent binding provides an irreversible attachment that is required for some application such as coating of implants or microarrays because the ligand should not dissociate or exchange with other compounds [62, 85]. Covalent immobilization of some antibacterial ligands on thiol/gold SAMs is presented in Table 4.

## 5. Nanostructured SAMs: Trend for Dental Implants

**Figure 5:**
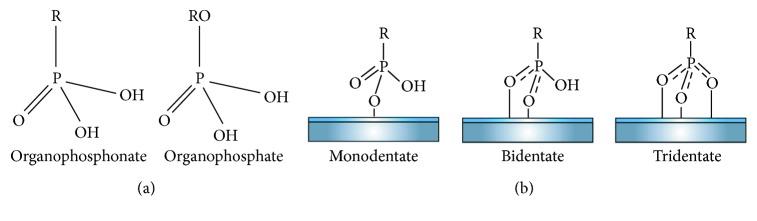
(a) Structure of organophosphate and phosphonate compounds. Adapted from Durmaz [127]. (b) Different bonding modes of a phosphonate unit to a metal oxide surface [130].

As described previously, SAMs of alkanethiol on gold are the most used, but the formation of well-ordered and strong alkanethiol monolayers has been extremely limited on metal oxides such as the titanium since alkanethiols generally do not adhere to metal oxides or are easily removed by rinsing. Among the self-assembled organic molecules, organophosphorus compounds are somewhat less often characterized compared to thiols but are becoming of great practical interest because of their ability to produce SAMs on a range of metal oxide surfaces including titanium [127, 128]. They also have attracted interest as an alternative to organosilane compounds in the functionalization of metal oxide surfaces due to the large number of available organophosphorus functional molecules and because the reaction mechanisms are not water sensitive [127, 129]. As indicated in Table 3, organophosphorus compounds for SAMs can be of organophosphonates (or phosphonic acids) and organophosphates (or phosphate ester), being structurally identical. An organophosphate has 4 oxygens with an alkyl group connected via a phosphoester bond, while organophosphonates have 3 oxygens with a carbon attached directly to phosphorus (Figure 5). The lack of a hydrolysable P-O-C linkage makes phosphonate compounds more stable in aqueous solution and easier to make SAMs than organophosphate compounds. Phosphonates and phosphonic acids form SAMs on TiO_2_ surfaces by the formation of Ti-O-P bonds [127].

The reaction of long-chain alkylphosphonic acids with metal oxide leads to dense, well-ordered SAMs [76, 131] that can find applications in a wide range of fields such as catalysis, corrosion resistance, microelectronics, chemical sensors [127], and in biomedical field, particularly in dental biomaterials for implants and orthodontic appliances. Like alkanethiol on gold and phosph(on)ate metal oxide, SAMs form monolayers with a “tail-up” orientation and a tilt angle of the hydrocarbon chains of about 30° with respect to the surface normal [128]. The binding mode of organophosphorus molecules has been proposed to be mono-, bi-, and tridentate (Figure 5), which is dependent on both the surface (i.e., titanium) and the nature of the organophosphorus compounds (i.e., phosphonate or phosphate), and being bidentate for titanium [132].

Gawalt et al. [133] have reported that self-assembly of alkanephosphonates on the native oxide surface of Ti can be affected by a simple procedure of aerosol deposition of octadecylphosphonic acid followed by solvent evaporation with subsequent heating at 120°C for 18 h giving strongly surface-bound, ordered films of the alkanephosphonate species, which resist removal by solvent washing or mechanical peel testing. Hähner et al. [134] studied the adsorption of octadecyl phosphate (ODP) onto several oxide surfaces including titanium, showing densely packed SAMs with the packing density analogous to that of alkanethiols on gold. Clair et al. [135] studied and compared the assembly of dodecylphosphoric acid (DDPA) on polished and on nanotextured titanium disks. After immersion on DDPA, smooth Ti surfaces presented a water contact angle of 88°, demonstrating successful deposition of a hydrophobic molecular film, and an average thickness of 20 Å, suggesting that a monolayer of material was deposited (the theoretical length of straight molecules is 18 Å). However, nanotextured Ti surfaces presented a greater hydrophobicity because of its nanoroughness, with contact angle as high as 120°, which is higher than that in an ideally flat surface. Due to the difference between the molecular height (2 nm) and the substrate average pit size (20 nm), the binding behavior of DDPA molecules is expected to be similar on smooth and nanotextured surfaces; considering the fact that previous studies showed that alkanephoshoric acid forms only monolayers on titanium, the authors assumed a similarity in the formation of a monolayer on this (nanorough) surface. Infrared spectroscopy for a flat surface provides characteristic methylene group peaks (CH2_υasymm_ 2933 cm^−1^ and CH2_υsymm_ 2858 cm^−1^) while the nanotextured surface presented peaks shifted (CH2_υasymm_ 2924 cm^−1^ and CH2_υsymm_ 2854 cm^−1^), with these differences in frequencies reflecting physical states of the phosphate monolayer on the surface, from a relatively densely packed phase on nanotextured Ti to a low-density disordered film on flat-polished Ti. In addition, some deterioration of the hydrophobic properties of the films was observed after 20 days in air and 10 days in buffer solution without further degradation after an additional storage for 1 month in ambient air. However, prolonged exposure of the samples (3 weeks) to the buffer solution resulted in a significant desorption of the organic film. The authors emphasize that alkanephosphoric acid films are relatively resistant to aging in a physiological-like environment when compared to thiol-based SAMs, in which spontaneous desorption occurs within a few days of immersion in various solvents, and that a further increase of their durability should be possible by optimizing film properties (e.g., by using longer alkyl chain molecules which should produce better molecular packing in the film). Spori et al. [136] reported the influence of chain length on phosphate SAMs showing a higher degree of order and packing density within the monolayers with alkyl chain lengths exceeding 15 carbon atoms forming crystalline structures and with an average alkyl chain tilt angle of 30° to the surface normal, similar to thiol/gold system. Lecollinet et al. [137] studied the adsorption of a monolayer of five bisphosphonates on oxidized surfaces of titanium and stainless steel. The authors highlighted that the molecule having a perfluoropolyether linked to a bisphosphonate moiety can resist harsh conditions, such as lasting water immersion at 50°C for 6 months, at different pHs, autoclaving at 121°C, and biocorrosion required for dental application.

However, in those studies and others described below for immobilized bioactive molecules [138–147], the amphiphile adlayers were produced from solutions of alkanephosphonates in organic solvents, which can reduce the biocompatibility of the surface [127, 130]. Tosatti et al. [148] applied aqueous phosphates to titanium oxide and titanium metal films to serve as smooth, flat model surfaces, and a special titanium dental implant surface with a rough, highly corrugated surface. XPS showed to form densely packed SAMs onto titanium not only as flat surface but also for high surface area materials, such as the SLA dental implant surface, with the phosphate headgroups attaching to the titanium (oxide) surface and the terminal end group (either methyl or hydroxy) pointing toward the ambient environment (air, vacuum, or water). The authors point out that “The technique of spontaneous organization of organophosphate molecules on titanium (oxide) surfaces from aqueous solution is believed to have potential for the modification of titanium-based medical implants and devices with the aim of tailoring the surface chemistry (chemical or biological functionalities), including groups such as poly(ethylene glycol), cell-adhesive peptides or growth factors.” Complementarily, Zwahlen et al. [149] showed that dodecyl phosphate adsorbed from aqueous solution formed SAMs of comparable quality to those of the longer octadecyl phosphate prepared from organic solvent and those of similar thiol/gold systems. Therefore, such mixed SAMs on metal oxide surfaces are of particular interest to the biosensor and biomaterial field, because they allow tailoring surface properties in a precise manner and may prove to be highly relevant for controlling the interaction between the SAM-modified surface and biological systems, such as proteins, antibodies, and cells.

### 5.1. SAMs for Bioactive Osseointegration on Titanium

Viornery et al. [150] firstly showed the formation of a chemical link between Ti disks and three phosphonic acids in water. The bioactivity of the modified Ti disks was evaluated by incubating these disks in a physiological solution (Hank's balanced salt solution (HBSS)) for 1, 7, and 14 days. Modified surfaces showed only slightly higher calcium levels in the XPS analysis compared to the reference Ti surface, with the surface modified with ethane-1,1,2-triphosphonic acid (ETP) inducing the highest calcium phosphate deposition after 14 days incubation [150]. Afterwards, these same types of phosphonic acid-modified titanium disks were evaluated *in vitro* related to the proliferation, differentiation, and protein production of rat osteoblastic cells (CRP10/30) [151]. No statistical differences were found in osteoblast proliferation among the phosphonic acid-modified titanium, unmodified titanium, and tissue culture plastic (used as a positive control), indicating that the phosphonic acids used were not cytotoxic to the osteoblasts. For all surfaces evaluated, the alkaline phosphatase activity was comparable as negative control (tissue culture plastic). However, the total amount of protein, and especially the collagen type I synthesis, was sensitive to surface modification. On titanium modified with ETP, the total amount of synthesized protein was significantly higher than the titanium control surfaces [151]. Then, the authors stated that “The covalently attached phosphonic acid molecules on the Ti-metal surface thus may form a scaffold for new bone formation, ultimately leading to bonding of the implant to the host tissue.”

A different strategy to enhance titanium osseointegration was explored by Liu et al. [145] firstly through the introduction of different end groups including hydroxyl, carboxylic acid, phosphate, and vinyl via the formation of alkyl-based SAM on a Ti foil. Accordingly, a hydroxyapatite coating was successfully obtained with phosphate and carboxylic acid after soaking the Ti foil in a solution that contained ions at a concentration 1.5 times higher than SBF. Then, these authors [146] investigated hydroxyapatite formation on Ti surfaces with various end groups, demonstrating that carboxylic acid as an end group provided the optimal SAM surface for nucleation and growth of biomimetic crystalline HA. It seems that the affinity of carboxylic acid for nuclei of CaP plays a pivotal role in the promotion of HA crystallization at surface. In addition to the functional groups, alkyl chain length of phosphonic acids should also be considered as a factor that influences hydroxyapatite crystallization at surface. Next, Wu et al. [147] evaluated the deposition from simulated body fluid of CaP onto carboxylic acid-terminated phosphonic acid SAMs with three different lengths of an alkyl chain (3, 6, and 16 methylene units) to compare the ability of promoting CaP crystallization. SEM, XPS, and X-ray diffraction revealed that the formation of PA SAMs accelerates the deposition of poorly crystallized HA in an alkyl chain length-dependent manner, primarily due to the higher surface density of Ca^2+^-attracting carboxylic acids. Among PAs studied here, PA containing a 16-carbon alkyl chain gave rise to the titanium surface most effective for the deposition of hydroxyapatite.

Gawalt et al. [138, 139] reported that alkylphosphonic acids (11-hydroxyundecylphosphonic acid) self-assemble on the native oxide surfaces of Ti and Ti6Al4V, followed by a heating step that binds the acids strongly to these surfaces as ordered phosphonate films. These SAMs with OH-terminated groups were activated to a maleimide group and then immersed in an aqueous solution of cell-adhesive peptide RGD-cysteine. The adhesion and spreading of the osteoblasts on RGD-modified Ti surface were quite substantial after 24 h and even more so after 3 days. Indeed, cell proliferation continued unabated throughout the test period on this surface. The morphology and actin cytoskeleton of cells were observed by staining with rhodamine phalloidin, with cells remaining small and rounded with no organized actin cytoskeleton on control substrates. However, more than 90% of cells adherent to RGD-modified substrates became well spread and organized their actin filaments into robust stress fibres. Danahy et al. [140] constructed more complex SAMs from *α*,*ω*-diphosphonic acids self-assembled on the native oxide surfaces of Ti and Ti6Al4V and thermally treated to get strongly bonded phosphonate monolayers. Data from infrared and X-ray spectroscopies and water contact angle showed that the films bind to the surface by one phosphonate unit while the other remains free as a phosphonic acid. Then, the SAMs were treated with zirconium tetra(tert-butoxide) to give surface Zr complex species. Finally, these surface-bound alkoxides can be further derivatized with the insertion of maleimide group followed by binding the RGD-cysteine peptide. Surfaces modified with RGD were stable to hydrolysis under physiological conditions and mechanically strong and shown to be effective to promote osteoblast adhesion and proliferation with organized actin filaments and vinculin-positive focal adhesions.

Adden et al. [141] used two different phosphonic acid monolayer films for immobilization of bioactive molecule BMP2 on titanium surfaces. Monolayers of (11-hydroxyundecyl) phosphonic acid and (12-carboxydodecyl) phosphonic acid molecules were produced by a simple dipping process and the terminal functional groups on these monolayers were activated (carbonyldiimidazole for hydroxyl groups and *N*-hydroxysuccinimide for carboxyl groups) to bind amine-containing molecules as the BMP2. The hydroxyl-terminated SAM is better ordered and orientated than the carboxyl-terminated SAM, and the CDI-activated surfaces (OH-terminated SAMs) gave higher amounts of BMP2 bound than the NHS-activated surfaces (COOH-terminated SAMs).

A different bioactive SAMs coating strategy was done by Mani et al. [152, 153] with the use of OH-terminated phosphonic acid SAM on Ti prepared from aqueous solution followed by the chemical attachment of a model drug flufenamic acid through three different methods of esterification (acid chloride esterification, dry heat esterification, and direct esterification). The drug release profiles of TSAMs prepared via acid-chloride esterification exhibited large data scatter, probably because the drug molecules were not uniformly attached to SAM-coated metal surfaces while TSAMs prepared by dry heat and direct esterification methods showed an initial burst release of the drug followed by a sustained slow release for up to 2 weeks. Thus, this study suggests “the potential for using SAMs as an alternate system for delivering drugs from coronary stents and other metal implants” [152]. Then, Mani et al. [153] used SAMs with flufenamic acid only attached by direct esterification to study their interaction with human aortic endothelial cells (HAECs), showing that the adhesion of HAECs on TSAMs was equivalent to that of control metal surfaces and superior to that of plain glass surfaces with the cells continued to proliferate on TSAMs even though the rate of proliferation was slower than plain glass or control-Ti. Moreover, the spreading of HAECs on TSAMs with typical polygonal shape indicated that these surfaces are conducive to endothelialization. The expression of surface adhesion protein (platelet endothelial cell adhesion molecule-1) on TSAMs indicated that the endothelial cells preserved their phenotype on these surfaces. Thus, this study demonstrated that TSAMs do not elicit an adverse response from endothelial cells in *in vitro* conditions.

Recently, Rudzka et al. [154] modified cpTi surfaces by producing mixed and patterned SAMs in order to induce hydroxyapatite nucleation and growth for bone tissue engineering. Mixed-SAMs were prepared from aqueous solutions having different fractions of 11-hydroxyundecylphosphonic acid (UDPA, -OH terminal group) and 12-phosphonododecylphosphonic acid (PDDPA, -PO(OH)_2_ terminal group) and patterned-SAMs from single THF solutions of 16-phosphonohexadecanoic acid (PHDA, -COOH terminal group) and octadecylphosphonic acid (ODPA, -CH_3_ terminal group) followed by laser ablation. These authors have shown that the PHDA-SAMs without laser treatment promote significantly the hydroxyapatite formation with smaller clusters, demonstrating that the presence of carboxyl groups on the cpTi surface is more favorable for the hydroxyapatite nucleation and growth in SBF than on the laser-ablated surface.

Rojo et al. [155] used a simple, effective, and clean methodology through the self-assembly chemisorption onto Ti6Al4V alloy surfaces of alendronate, which is a well-known bisphosphonate commonly used in osteoporosis therapy and treatment of other bone diseases. XPS spectroscopy revealed that an effective mode of bonding is created between the metal oxide surface and the phosphate residue of alendronate, leading to the formation of homogeneous drug distribution along the surface. In addition, *in vitro* studies showed that alendronate SAMs induce differentiation of hMSC to a bone cell phenotype and promote bone formation on modified surfaces, as evidenced by upregulation in the expression of early markers of osteogenic differentiation (Runx2, osteopontin (OPN), alkaline phosphatase (ALP), and BMP2).

### 5.2. SAMs with Antiadhesive and Antibacterial Properties on Titanium

Byun et al. [156] synthetized a PEG-phosphonic acid terminated with an amino group (PA-C11-EG3-NH2) that is used to make SAMs onto titanium surface by aqueous immersion. This denominated pSAM was sequentially modified by EMPSA conjugation through EDC/NHS chemistry to insert a terminal thioester functional group followed by PEGylation through NH2-Cys-PEG. Ellipsometry, goniometry, and XPS unambiguously confirmed the presence of PEGs, which provided nonfouling effects of surfaces, preventing the biological adhesion of cells as the NIH-3T3 adhered cells were reduced by 92.3% after PEGylation.

Amalric et al. [142, 143] developed antibacterial nanocoatings on titanium and stainless steel through the functionalization of phosphonate monolayers of mercaptododecylphosphonic acid (MDPA) with silver nitrate (AgNO_3_) by a two-step scheme: (i) deposition of a thiol-functionalized monolayer by reaction with MDPA solution and (ii) reaction of the terminating thiol groups with silver nitrate to form silver thiolate species. Thiol-terminated groups are expected to react readily with silver cations to form silver thiolates with high formation constants and, accordingly, the silver thiolate groups should be stable toward hydrolysis, but silver ions can be selectively released by exchange between the silver thiolate groups at the surface of the monolayers and the free thiol groups exposed at the surface of the bacterial membrane proteins, with the reaction of Ag+ ions with thiol groups in the bacterial membrane proteins playing an essential role in bacterial inactivation. FTIR confirmed the binding of MDPA to the surface on both the titanium and the stainless steel, suggesting the formation of moderately ordered monolayers compared to alkylphosphonic acid monolayers deposited on similar substrates. XPS analysis confirmed the effectiveness of these surface modifications. Postmodification with AgNO_3_ resulted in the conversion of most of the terminal thiol groups into silver thiolate species, which represented about 60% of all sulfur species in the final samples, with the density of silver at the surface estimated to 3.5 ± 1 Ag·nm^−2^, corresponding to about 0.6 nmol·Ag·cm^−2^. Thus, the amount of silver was very low compared to other antibacterial silver-coated materials reported in the literature, such as the silver content in stainless steel or titanium samples modified by ion implantation or by physical vapor deposition ranging from 80 to 1700 nmol·Ag·cm^−2^. Despite their very low silver content, MDPA + AgNO_3_ monolayers strongly decreased the bacterial adhesion of the surface compared to the bare titanium or stainless steel substrates: a 3- to 5-log reduction in the number of viable adherent bacteria was found for the four bacterial strains tested (*E. coli*, *S. aureus*, *S. epidermidis*, and *P. aeruginosa*). Furthermore, the antibacterial efficiency of MDPA + AgNO_3_ monolayers remained excellent even after incubation for 3 days at 37°C in fresh human blood plasma, with a 4-log reduction of the number of viable adherent bacteria on the coated substrate compared to unmodified substrate. The MDPA + AgNO_3_ coating deposited on titanium or stainless steel also strongly decreased the density of bacterial biofilm formed after incubation for 3 days in a culture of *E. coli*, *S. epidermidis*, or *P. aeruginosa*. In addition, the growth of *E. coli* biofilm on titanium modified by MDPA + AgNO_3_ was significantly inhibited for about 1 week. Even more, since the release of silver ions by hydrolysis of the silver thiolate groups was negligible, the antibacterial effect observed in this study could result from the exchange of silver between the thiolate groups at the surface of the MDPA + AgNO_3_ monolayers and the free thiol groups exposed at the surface of the bacterial membrane proteins. Antibacterial transitory effect was obtained for an extremely low Ag content compared to conventional coatings, which is important to avoid any toxicity issue and to minimize the release of silver in the environment, which could facilitate the selection of resistant strains. Rather, the aim of these coatings is to prevent contamination during handling of the implant and surgery, then for the first few days after the implantation, which are considered critical. Then, Tîlmaciu et al. [144] showed that these SAMs on titanium significantly inhibited *E. coli* and *S. epidermidis* adhesion and biofilm formation *in vitro*, while allowing attachment and proliferation of MC3T3-E1 preosteoblasts. Moreover, osteogenic differentiation of MC3T3 cells and murine mesenchymal stem cells was not affected by the nanocoatings. Sterilization by ethylene oxide did not alter the antibacterial activity and biocompatibility of the nanocoatings. After subcutaneous implantation of the materials in mice, MDPA + AgNO_3_ nanocoatings exhibit significant antibacterial activity and excellent biocompatibility, both *in vitro* and *in vivo*, after postoperative seeding with *S. epidermidis*.

Vaithilingam et al. [157] immobilized Ciprofloxacin® (an antibacterial drug) to a carboxylic acid-terminated phosphonic acid based self-assembled monolayers (SAMs) adsorbed on a selectively laser melting (SLM) Ti6Al4V structure by immersion deposition method in THF. Ciprofloxacin-coated Ti6Al4V surfaces are highly stable under the oxidative ambient laboratory conditions for 1, 2, 4, and 6 weeks. When immersed in 10 mm of Tris-HCl buffer (pH 7.4), the drug was observed to release in a sustained manner with 50% of the drug released after 4 weeks and approximately 40% of the drug remaining after 6 weeks. Antibacterial susceptibility tests revealed that the immobilized drug was therapeutically active upon its release against *S. aureus* and *E. coli*. Then, these authors also used the same strategy to immobilize paracetamol, which was stable for over four weeks and then began to desorb from the surface, showing a potential to improve biocompatibility and reduce surgical complications after implant placement [158].

### 5.3. SAMs with Dual Biofunctions

Kang et al. [28] coated titanium with a nonbiofouling poly(poly(ethylene glycol) methacrylate) (pPEGMA) by surface-initiated polymerization to Br-terminated SAMs of phosphonic acid, and BMP2 was chemically conjugated to the activated pPEGMA films by DSC/DMAP chemistry. The BMP2-conjugated pPEGMA films induced adhesion and differentiation of mesenchymal stem cells.

Moreover, Gnauck et al. [159] synthetized a carboxy-terminated oligo (ethylene glycol) alkanephosphate with the OEG for resistance against nonspecific protein adsorption and cells/bacteria adhesion and the COOH-terminal functional group as a linker for the attachment of specific bioligands, such as peptides and proteins to be present at the surface. XPS data showed that the monomolecular layer is attached with the phosphate group to the substrate but not fully ordered or taking an all-trans conformation. However, this study did not present any result concerning protein adsorption or cell adhesion with only chemical and structure characterization. Bozzini et al. [160, 161] synthesized PEG-terminated alkanephosphate that was codeposited with OH-terminated alkane phosphates from aqueous solution onto TiO_2_ films. XPS and ellipsometry of the resulting mixed SAMs indicate that the PEG density can be controlled by varying the mole fraction of PEG-terminated phosphates in the solutions used during the deposition process, leading to surfaces with different degrees of protein resistance [160] and reduction of bacterial adhesion (*S. mutans*): As the PEG surface density increased, the protein adsorption and bacterial adhesion considerably decreased when compared to uncoated titanium surfaces, while maintaining osteoblast proliferation up to 7 days of culture *in vitro* with the greatest levels of metabolic activity at the highest PEG surface concentrations [161]. These results are extremely promising in view of a potential clinical application in dental implants, where reduction of bacteria adhesion and stimulation of bone formation are both highly desirable.

Other approaches using nonphosphate SAMs have been reported, showing results oriented to improve the osseointegration and reduce the bacterial adhesion [162–165].

## 6. Conclusions

The present review shows that dental-based implant therapy after 30 years is a predictable short-term treatment to patient with full or partial edentulism. However, long-term success and survival of implant need more research looking for a more stable interface tissue/implant. Nanoscale modifications of surface implants have been an active scientific area, where new approaches such as SAMs are providing strategies to modulate tissue response and microbiota microenviroment in terms of bioactivity, antiadhesion, antibacterial, or combined effects.

- Titanium with SAMs for bioactive osseointegration effect has been highly studied with several strategies such as exposure of carboxylic terminal to promote calcium phosphate or hydroxyapatite deposition, with immobilized cell adhesive RGD peptides to induce osteoblast attachment, spreading, and proliferation and with immobilized bone morphogenetic proteins (BMPs) to promote bone formation.
- Antiadhesive and antibacterial SAMs on titanium have been sparsely worked with few studies, based on monolayers with protruding group of ethylene glycol and in the immobilization of metals ions and molecules with activity against bacteria, respectively.
- SAMs on titanium with combined bioactivity and antiadhesion or antibacterial effect have been little described with a monolayer of ethylene glycol to which pro-osseointegration molecules might be immobilized.

## Figures and Tables

**Table 1:** Bioactive osseointegration, antiadhesive, and antibacterial coatings on titanium.

Surface	Coating type	Molecule	Study type	Effect	Ref.
Bioactive osseointegration	Covalent immobilization of osseointegration molecules	RGD peptide	*In vitro*	Supports osteoblast attachment and spreading, and significant mineralization after 14 and 21 days	[21]
			*In vitro*	Significantly improves the osteoblast adhesion, proliferation, and alkaline phosphatase (ALP) activity while retaining high antibacterial efficacy after aging for 21 days in PBS	[22]
			*In vivo*	Significant increase in bone formation after 4 weeks *in vivo* in rat femurs and in a rabbit model	[29, 30]
		BMP4	*In vitro*	Induces significant alkaline phosphatase activity in pluripotent C3H10T1/2 cells	[24]
		BMP2	*In vitro*	Only 8% of the immobilized BMP2 seems to be available for interaction with the cells and able to induce the signaling cascade with cytocompatible for C3H10T1/2 cells	[31]
			*In vitro*	Reduces the bacterial adhesion (*S. aureus* and *S. epidermidis*) and significantly promotes attachment, alkaline phosphatase activity, and calcium mineral deposition of both osteoblast and human bone marrow-derived mesenchymal stem cells	[20]
			*In vivo*	Titanium screw implants with nano-anchored oligonucleotides strands hybridized with conjugated rhBMP2 exhibited enhanced bone ingrowth into the perforations and increased bone implant contact after 1 and 4 weeks compared to controls. No difference was seen after 13 weeks. Bone density around the outer implant surface did not differ significantly at any of the intervals. Therefore, rhBMP2 immobilized on the surface of titanium implants through nanoanchored oligonucleotide strands can enhance bone implant contact	[32]

Antiadhesive	Polymer coating	PEG	*In vitro*	Inhibits salivary protein adsorption and the attachment of *S. gordonii* and *S. mutans* biofilm was easier to be detached	[25]
		PLL-g-PEG	*In vitro*	Human serum adsorbed was below the detection limit of the optical sensor technique (OWLS) (<1-2 ng/cm^2^). Reduces fibrinogen adsorption by 96%. Decreases *S. aureus* adhesion by 89–93%	[33, 34]
		PLL-g-PEG conjugated to catechols groups^1^	*In vitro*	Suppresses fibrinogen adsorption. Resists attachment of the cyanobacterium *Lyngbya* spp. for at least 100 days	[35]
		PEG conjugate to catechols groups	*In vitro*	Resistant to serum proteins (<1 ng/cm^2^ to detection limit of OWLS). Almost free of blood protein adsorption. No cytotoxicity against bone-marrow stem cells. Reduces 95% of serum protein adsorption	[36–38]
	—	Multivalent PEGylated peptides	*In vitro*	90% PEGylated peptides remain in surface. 90% reduction in *S. aureus* biofilm	[39]

Antibacterial	Metal ion incorporation	Silver	*In vitro*	Activity effects against periodontal and peri-implant pathogens, including *P. gingivalis*, *P. intermedia*, *A. actinomycetemcomitans*, *F. nucleatum*, *Tannerella forsythia*, and *S. aureus*	[40, 41]
			*In vitro*	Limits antibacterial activity against *P. gingivalis* and *A. actinomycetemcomitans*, probably due to the formation of silver compounds such as AgCl, Ag_2_O, and Ag_2_S	[42, 43]
			*In vitro*	Improves the antibacterial effect against *S. aureus* and preserves human gingival fibroblasts viability	[44]
			*In vitro*	Very strong (greater than 4-log or 99.99% reduction) antibacterial effect against *P. aeruginosa* for 24 h. Osteoblast adhesion, spread, and proliferation higher than bare-Ti and so does not cause cytotoxicity	[45]
		Zinc	*In vitro*	No antibacterial activity against the periodontal bacteria *P. gingivalis* and *A. actinomycetemcomitans*, possibly due to the formation of zinc halogens, oxides, or sulphides	[43]
		Copper	*In vitro*	Antimicrobial effect on *E. coli* and especially on *S. aureus*, with the lethal concentration for *S. aureus* of 5 µg/ml	[46]
			*In vitro*	Antibacterial activity (90%) against *S. aureus*	[47]

—	Biocidal release	Gentamicin into a degradable PDLLA	*In vivo*	Animals receiving systemic therapy alone had a recovery rate of about 15%, whereas animals receiving the gentamicin-coated implants had an 85% recovery rate. Human patient with infection-free after 1 year and no gentamycin levels in blood	[48]
		Mixtures of antibiotics or antiseptics into PLLA	*In vitro* and *in vivo*	Effective in eliminating *S. aureus* infection without cytotoxic effects	[49]
		Chlorhexidine into PLLA and politerefate	*In vitro*	Concentration of chlorhexidine remained at therapeutic levels for 200 h (8 days) before disappearing completely. Cytocompatible to hTERT fibroblast cells	[50]
		Gendine (chlorhexidine + gentian violet)	*In vitro*	Active against methicillin-resistant *S. aureus* (MRSA), preventing the formation of biofilm (90% reduction)	[51]
		Vancomycin into silica sol-gel thin film	*In vitro*	Releases drug above the MIC and degrades after about 2 weeks *in vitro*	[52]
		Minocycline and rifampicin	*In vivo*	In a rabbit model that induced infection by inoculating *S. aureus* in the femoral medullary canal, the coated implants had an infection rate of 38% compared with 100% for the noncoated	[53]

—	Covalent immobilization of biocidal	Vancomycin	*In vitro* and *in vivo*	Strong bactericidal activity against *S. epidermidis* and *S. aureus* over long periods of time (up to 6 weeks) *in vitro*. Stable bactericidal activity and reduced infection rates when implanted in an infected rat model	[54]
		Vancomycin conjugated to PEG-anachelin	*In vitro*	Only dead cells (*B. subtilis*) were detected on surface	[55]
		Gentamicin and penicillin	*In vitro*	The covalently immobilized antibiotics retain the antibacterial properties as indicated by a significant reduction in the viability of contacting *S. aureus*	[56]
		AMP Tet213	*In vitro*	Activity against both Gram-positive (*S. aureus*) and Gram-negative (*P. aeruginosa*) bacteria with 10^6^-fold reductions of both bacterial strains within 30 min	[57]
		AMP GL13K	*In vitro*	Significantly fewer live cells of *P. gingivalis* than disks coated with a control peptide and uncoated Ti under static culture conditions. This GL13K coating showed to be cytocompatible by an adequate proliferation of osteoblasts and human gingival fibroblasts. Kills bacteria and prevents formation and growth of *S. gordonii* biofilms in a drip-flow bioreactor and under regular mild-agitation conditions, with rupture of the cell wall	[58, 59]
		Tet-20	*In vitro* and *in vivo*	Excellent activity against Gram-negative *P. aeruginosa* and Gram-positive *S. aureus*, as well as biofilm resistance *in vitro*. The coating had no toxicity to osteoblast-like cells and showed insignificant platelet activation and adhesion, and complement activation in human blood. Protects bacterial infection *in vivo* (rat model) against infected *S. aureus*	[60]
		hLf1-11	*In vitro*	Reduction in bacterial adhesion, early-stage biofilm formation, and growth of planktonic of *S. sanguinis* and *L. salivarius*	[61]

**Table 2:** Analytical capabilities of commonly used techniques for SAM characterization. Adapted from Liedberg and Cooper [67].

Experimental technique	Analytical capability							
	Thickness	Interfacial tension	Coverage	Chemical composition	Orientation of molecule or group	Alkyl chain density	Defects and their distribution	Roughness chemical homogeneity
Ellipsometry	++	−−	0	−−	−−	0	−−	0
Contact angle goniometry	−−	++	−	0	+	0	−	+
Cycle voltammetry	−	−−	++	−−	−−	++	++	−−
Infrared spectroscopy	+	−	+	+	++	++	−	−−
XPS	0	−−	++	++	+	0	−−	−−
QCM	+	−−	++	−−	−−	0	−−	−−
AFM	−−	0	+	−	−	−	++	++

Analytical capability: ++, excellent; +, good, 0, fair; −, poor; −−, not applied.

**Table 3:** Types of SAMs according to the surface.

Surface		Surface active head group		Ref.
Noble metals	Gold, silver, copper, platinum, and palladium	Organosulfur compounds	Alkanethiols (R-SH), dialkyl sulfide (R-S-R), dialkyl disulfide (R-S-R)	[62, 64, 68–73]
Hydroxylated surfaces	Silicon oxide/silica (SiO_2_), aluminum oxide (Al_2_O_3_), quartz, glass, and mica	Organosilanes or organosilicon derivatives	Alkylchlorosilanes (R-Si-Cl_3_), alkylalkoxysilanes (R-Si-(OCH_3_)_3_), and alkylaminosilanes (R-Si-(NHCH_3_)_3_)	[64, 65, 74]
Metal oxide	Silver oxide, aluminum oxide (Al_2_O_3_), zirconium dioxide (ZnO_2_, zirconia), titanium/titanium oxide (TiO_2_), and native oxide stainless steel	Carboxylic acids	*n*-Alkanoic (carboxylic) acids (C_n_H_2*n*+1_COOH)	[64, 74, 75]
		Organophosphorus compounds	Phosphates (RPO_3_^2−^), phosphonates/phosphonic acids (RP(O)(OH)_2_)	[65, 74, 76–79]

**Table 4:** Bioactive osseointegration, antiadhesive, and antibacterial gold SAMs.

Surface	Strategy	SAMs terminal group	+Ligand	Effect	Ref.
Bioactive osseointegration	Covalent binding of osseointegration molecules	Maleimide	CGGRGDS-NH2	Efficient and specific attachment of 3T3 fibroblasts	[108]
		Chloracetylated	Ac-CGGGRGDSP-NH_2_	Fibroblast adhesion and spreading	[109]
		Azide (click chemistry reaction)	RGDSP	Minimal nonspecific protein adsorption (lysozyme and proteins in fetal bovine serum) and selective adhesion and spreading of human mesenchymal stem cells (hMSC). Moreover, RGDSP intermolecular spacing of 36 nm or less (≥0.01 mol% on the surface) is sufficient for hMSC adhesion and a spacing of 11 nm or less (≥0.05 mol% on the surface) is sufficient for cell spreading and focal adhesion complex formation	[110]
		Phosphonates	Engineered fusion protein comprising cutinase and sections of fibronectin (FnIII10)	Leaves the cutinase bound to the surface, but the attached protein extends into the ambient solution with a defined orientation. Substrates presenting cutinase-FnIII_10_-mediated rapid attachment and spreading of Swiss 3T3 fibroblasts, while substrates presenting cutinase or the phosphonate ligand alone did not support cell attachment	[111]
		Hydroquinone^5^	*RGD* cyclopentadiene	Promotes Swiss 3T3 fibroblasts attachment, spreading, and migration from surface with adsorbed fibronectin to immobilized RGD	[112]

Antiadhesive	—	Ethylene glycol, HS(CH_2_)_11_(OCH_2_CH_2_)_*n*_OH (EG_*n*_, *n* = 3–7 or OEG)	—	Low adsorption of several blood proteins such as albumin, heparin, and thrombin as well as blood cell adhesion of leukocytes and platelets	[92, 93, 113–116]
				Prevents *H. pylori* adhesion and significantly reduces the viability of adhered bacteria	[86]
				SAMs prepared with latent aldehyde and OEG terminal showed high protein resistance (IgG) and ability to efficiently bound small bioligands or small heterobifunctional crosslinkers with hydrazide functions to the aldehyde functions on the SAM	[117]
				SAMs prepared with anhydride having H_2_N(EG)_*n = 3 − 6*_*-*H resist nonspecific protein adsorption (fibrinogen, lysozyme, and ribonuclease A) similar to a single-component SAMs involving chemisorption of OEG-terminated alkanethiols on gold	[118]
				Mixed SAMs (HS-EG6-COOH + HS-EG3-OH) resist to adsorption of cytochrome c and lysozyme	[119]
				Biotin-containing SAMs having an ethylene glycol background improve the selectivity to streptavidin, by avoiding nonspecific protein adsorption, and to subsequent biotin-labelled molecules with a right surface orientation	[105, 120–122]
				A natural direct thrombin inhibitor (*boophilin*) was successfully expressed, purified, biotinylated, and immobilized on biotin SAMs, able to adsorb thrombin in a selective way to delay surface-induced plasma coagulation, and so be used for the development of novel hemocompatible materials for blood-contacting devices	[107]

Antibacterial	Covalent binding of antibacterial molecules	Anhydride^3^	*N*-*α*-Ac-L-Lys-D-Ala-D-Ala (*N*-R-Ac-KDADA)	Biospecific interaction of vancomycin with this fragment from the bacterial cell wall	[123]
		Free carboxylic acid^4^ (HS-EG6-COOH + HS-EG3-OH) + EDC/NHS chemistry	Anti-*E. coli* O157:H7	SPR biosensor with alkane monothiol surface was demonstrated to be very rapid, sensitive, and specific for potential application in detection of *E. coli* O157:H7	[124]
			Magainin I	Reduces by more than 50% the adhesion of bacteria (*L. ivanovii*, *E. faecalis*, and *S. aureus*) at the surface, together with the killing of the bacteria that nonetheless adhered to the surface	[125]
		Biotin	Biotin-*H. pylori* glycan structures	Several biotinylated adhesins specific to different strains of *H. pylori* were bound onto biotin SAMs showing that these immobilized ligands maintain an ability to specifically bind *H. pylori* and thus offering new insights into innovative strategies against *H. pylori* infection based on the scavenging of bacteria from the stomach using specific *H. pylori*-chelating biomaterials	[126]
